# Advancement in Stem Cell Therapy for Ischemic Myocardial Cell: A Systematic Review

**Published:** 2018-10-01

**Authors:** Syeda Beenish Bareeqa, Fazeela Bibi, Syed Ijlal Ahmed, Syeda Sana Samar

**Affiliations:** 1 Jinnah Medical and Dental College, Karachi, Pakistan; 2 Medical Graduate, Liaquat National Medical College and Hospital, Karachi, Pakistan; 3 Jinnah Sindh Medical University, Karachi, Pakistan

**Keywords:** Mesenchymal stem cells, Myocardial infarction, Cardiomyocytes, Hematopoietic stem cells

## Abstract

**Background: **Cardiac muscle possesses a limited capacity to regenerate its tissue on its own. It is less likely to reverse the altered cardiac functioning to its normal physiological state after a major myocardial infarction. Stem cell transplantation provided a unique therapeutic approach in managing such injuries. There has been a substantial debate about the complexity, scope and medical application of stem cell transplantation in past few years.

**Materials and Methods: **An extensive review of medical literature was conducted to establish the consensus about the possible mechanism of cell renewal, associated complications and risks of failure of this technique. Twenty cases of mammalian animals and twenty-four cases of stem cell transplantation in human subjects were reviewed.

**Results: **Most common associated complication was re-stenosis of coronary artery. Few clinical trials reported the failure in improving cardiac functioning. The success rate of stem cell transplantation was remarkable in the literature related to experimental animal subjects.

**Conclusion: **It was concluded that renewal of the cardiac cell is a result of induction of angiogenesis and prolonged cell survival. This topic still requires an immense amount of research to fill the gap in adequate knowledge.

## Introduction

 Recent classification of stem-cells has divided it into three generations. First generation includes skeletal myoblasts (SMs), bone marrow mononuclear cells (BMMNCs), hematopoietic stem cells (HSCs), endothelial progenitor cells (EPCs) and mesenchymal stem cells (MSCs). Second generation has cardiopoietic MSCs (cpMSCs), cardiac progenitor cells and induced pluripotent stem cells (iPSCs). Next generation therapy includes cell enhancement approaches and cell-free approaches^   1 ^. Mesenchymal stem cells or bone marrow cells are multipotent adult stem cells that have potential to differentiate into a variety of lineages^   2 ^. Cardiac tissue possesses a transient regenerative capacity which eventually ends up with scarring of damaged tissue. But MSCs are capable enough to differentiate into endothelial cells, vascular smooth muscle cells and perhaps even cardiac myocyte. Origin of the cells in the forming myocardium was determined by expression of EGFP. Its expression was combined with the labeling protein specific for myocytes, endothelial cells and smooth muscle cells. The percentage of new myocytes, endothelial cells and the smooth muscle cells expressing EGFP was 53% (n=7), 44% (n=7) and 49% (n=7), respectively^3^. 

A study conducted by Nagaya N et al. demonstrated in a rat model that after intravenous infusion, preferential site of attraction for MSCs were ischemic myocardial tissue. It differentiated into vascular endothelium cells and cardiomyocytes. The possible mechanism behind the reduction of infarct size, improved cardiac functions after acute MI and increment in capillary density was the induction of angiogenesis and myogenesis. It was believed that von Willebrand factor has a positive role in induction of new myocardial tissue^   4 ^. Biodistribution of MSCs depends upon the route of administration. Endocardial (EC), intracoronary (IC) and intravenous (IV) infusion are common courses to deliver stem cells. A study on porcine MI model concluded that IC and EC infusion of MSCs in post-MI subjects resulted in increased engraftment when compared with intravascular infusion^   5 ^.

Stem cell transplantation has been emerging as a new therapeutic intervention for different fields of medicine. But, a major dilemma in stem cell therapy for ischemic heart diseases is the low survival of transplanted cells in the peri-infarcted region. Number of pilot studies on MSCs transplantation demonstrated the failure of such intervention. Some studies reported the failure of transplantation within a week, while other reported that most implanted cells may die within 4 days after infusion into the ischemic heart. Electron microscopy revealed that dead cells had features of both irreversible ischemic injury and apoptosis ^6^^,^^7^ . 

The purpose of our study is to assess the advancements in stem cell transplantation, causes of its failure and the associated adverse outcomes. 

## MATERIALS AND METHODS

 Several different methods can be found in the vast literature on stem cell therapy, but only particular introduction methods of cardiac stem cell were highlighted in the literature from past few decades. Most common deployment techniques are introduction of stem cell via catheter or a 22-gauge needle which are minimally invasive techniques. However, different sites of implantation are found in literature review. 

In one study conducted in 2004, Shao-liang C et al. injected autologous mesenchymal cells into coronary artery along with normal saline. It was introduced 12 hours after PCI was done and showed much improvement in left ventricular function^   8 ^. A double-blind, randomized, controlled trial conducted in 2006 discussed 3 separate methods of MSC introduction in swine. Stem cell was delivered via IV infusion in ear vein catheter, intracoronary infusion after balloon inflation and an endocardial infusion into the infracted areas. IC infusion showed much promising results as compared to EC and IV methods^   5 ^. In another method discussed in 2003, transendocardial delivery of MSCs was performed using NOGA catheter after left heart catheterization^   9 ^. A prospective randomized study done in 2005 used novel epicardial technique to deploy autologous MSCs via 22-gauge needle. Autologous MSC tissue was extracted from iliac crest bone marrow^   10 ^_._ To the best of our knowledge, no non-invasive technique is currently present to transplant cardiac stem cells. 

## Results

 After the approval of this study from the Ethical Review Board of Jinnah Medical and Dental College, literature search was conducted on Pub-med, Embase and Google scholar on the literature which was published from January 1999 to January 2017. Details are given in Table 1 and Table 2. No limitation of age in human subjects and type of animal in mammalian subjects was established. The research was limited to humans and other mammalian animals. Only English medical literature was included in our review. The key MeSH and non-MeSH terms used were ‘Mesenchymal stem cells (MSCs)’, ‘Myocardial infarction (MI)’, ‘Cardiomyocytes’, ‘Hematopoietic stem cells (HSCs)’. 

**Table 1:** Reported cases of stem cell transplantation in animal subjects

Author/ Year of Publication	Animal Subject	Method of MI Induction	Type of Stem Cells Injected	Follow-up	Hemodynamic status	References
**Dai, W., et al/ ** **2005**	Rat	Left coronary artery ligation	MSCs	6 months	LVEF: improved After 4 week = 43.8% At 6 months= 41.8%	(35)
**Nagaya, N., et al/** **2004**	Rat	Left coronary artery ligation	MSCs	4 weeks	LVEF= improved About 53%	(4)
**Silva, G.V., et al/ 2005**	Dog	Left coronary artery ligation	MSCs	60 days	LVEF: improved 30 days after intramyocardial injection At rest= 48% With stress=27%	(36)
**Bel, A., et al/ 2003**	Sheep	Left circumflex artery	BM-MNC	8 weeks	LVEF= improved from 13% to 65%	(37)
**Davani, S., et al/** **2003**	Rat	Left coronary artery ligation	MPC	30 days	LVEF= improved About 43%	(38)
**Ghostine, S.d., et al/ ** **2002**	Sheep	Circumflex coronary artery catheterization	Skeletal myoblasts	1 year	LVEF= improved about 48%	(39)
**Jian-an, W., et al/ 2005**	Rabbit	Left coronary artery ligation	HMSCs	4 weeks	LVEF: improved Before operation=0.79+/- 0.07% After operation=0.66+/- 0.04 %	(40)
**Brasselet, C., et al/ ** **2005**	Sheep	Left coronary artery ligation	Skeletal myoblast	8 weeks	LVEF= improved From 47% to 54%	(41)
**de Silva, R., et al/ 2008**	Pig	LAD	BM-MNC	6 weeks	LVEF= improved From 24% to 29%	(42)
**Doyle, B., et al/ 2008**	Pig	Left circumflex artery	EPC	8 weeks	LVEF= improved about 49%	(43)
**Memon, I.A., et al/ ** **2005**	Dog	LAD	Skeletal myoblasts/BM- MNC	4 weeks	LVEF= improved About 47%	(44)
**Moelker, A.D., et al/ ** **2006**	Pig	Left circumflex artery	BM-MNC	4 weeks	LVEF= improved About 46%	(45)
**Tang, J., et al/ 2006**	Rat	Left coronary artery ligation	MSCs	4 weeks	LV function: improved LVSP=110mmHg LVEDP=13mmHg	(46)
**Berry, M.F., et al/ 2006**	Rat	Left coronary artery ligation	MSCs	8 weeks	LV function: improved Max left ventricular pressure=52mmHg	(47)
**Min, J.-Y., et al/ 2002**	Pig	Left coronary artery ligation	hMSCs & hfCs	6 weeks	LV function: improved At rest: LVSP=75mmHg LVEDP=10.4mmHg With stress: LVSP=36mmHg LVEDP=15mmHg	(48)
**Yang, Y., et al./ 2002**	Rat	Left coronary artery ligation	EDCs	6 weeks	LV function: improved LVSP=70% LVEDP=140%	(49)
**Tomita, S., et al/ 1999**	Rat	Cryoinjury to left ventricular free wall	BMCs	5 weeks	LV function: improved	(11)
**Sakakibara, Y., et al/ ** **2002**	Rat	Left coronary artery ligation	cardiomyocytes	4 weeks	LV function: no change LVEDP=9.4mmHg	(13)
**Nagaya, N., et al/ 2005**	Rat	Myocarditis induced by immunization with porcine cardiac myosin	MSCs	4 weeks	LV function: improved LVEDP=12mmHg	(12)
**Fujii, T., et al/ 2005**	Rat	Left coronary artery ligation	MNC AM AM-MNC	4 weeks	LVEDP: improved in AM- MNC only MNC=12mmHg AM=8mmHg AM-MNC=5mmHg	(50)

**Table 2:** Cases reported on human trials of stem cells transplantation

AUTHOR/ YEAR OF PUBLICATION	AGE	PRESENTING COMPLAIN	TYPE OF STEM CELLS INJECTED	FOLLOW-UP	PATIENT’S OUTCOME	INTERVENTION/ DRUG THERAPY	REFERENCE
**Stamm, C., et al/** **2003**	64	Anterior septum MI	AC133+ stem cells	10 months	SVT and pneumonia	PCI	(26)
**Stamm, C., et al/** **2003**	73	Infero-lateral MI	AC133+ stem cells	8.5 months	Bronchitis	PCI	(26)
**Stamm, C., et al/** **2003**	54	Inferior wall MI	AC133+ stem cells	7.7 months	None	PCI	(26)
**Stamm, C., et al/** **2003**	69	Anterior septum MI	AC133+ stem cells	6 months	Bleeding from left internal mammary artery	PCI	(26)
**Stamm, C., et al/** **2003**	75	Inferior wall MI	AC133+ stem cells	3.5 months	None	PCI	(26)
**Stamm, C., et al/** **2003**	55	Lateral wall MI	AC133+ stem cells	3 months	None	PCI	(26)
**Strauer, B.E., et al/ 2001**	46	Anterior septum MI	Autologous MSCs	10 weeks	None	PCI	(51)
**Patel, A.N., et al/** **2005**	56	Mild MI	CD34+ stem cells	6 months	None	PCI	(10)
**Patel, A.N., et al/** **2005**	76	Moderate MI	CD34+ stem cells	6 months	None	PCI	(10)
**Patel, A.N., et al/** **2005**	72	Mild MI	CD34+ stem cells	6 months	None	PCI	(10)
**Patel, A.N., et al/** **2005**	65	Mild MI	CD34+ stem cells	6 months	Hematoma at bone marrow harvest site	PCI	(10)
**Patel, A.N., et al/** **2005**	57	Moderate MI	CD34+ stem cells	6 months	None	PCI	(10)
**Patel, A.N., et al/** **2005**	61	Moderate MI	CD34+ stem cells	6 months	None	PCI	(10)
**Patel, A.N., et al/** **2005**	66	Mild MI	CD34+ stem cells	6 months	None	PCI	(10)
**Patel, A.N., et al/** **2005**	61	Mild MI	CD34+ stem cells	6 months	None	PCI	(10)
**Patel, A.N., et al/** **2005**	60	Moderate MI	CD34+ stem cells	6 months	None	PCI	(10)
**Patel, A.N., et al/** **2005**	74	Moderate MI	CD34+ stem cells	6 months	None	PCI	(10)
**Kang, H.-J., et al/ ** **2004**	64	Antero-lateral wall MI	Peripheral blood stem cells	6 months	None	PCI/Aspirin+ clopidpgrel, β-blockers, ACE inhibitors	(27)
**Kang, H.-J., et al/ ** **2004**	60	Anterior septum MI	Peripheral blood stem cells	6 months	Coronary artery restenosis	PCI/Aspirin+ clopidpgrel, β-blockers, ACE inhibitors, HMG CoA inhibitor	(27)
**Kang, H.-J., et al/ ** **2004**	67	Inferior wall MI	Peripheral blood stem cells	6 months	Coronary artery restenosis	PCI/Aspirin+ clopidpgrel, β-blockers, ACE inhibitors	(27)
**Kang, H.-J., et al/ ** **2004**	57	Antero-lateral wall MI	Peripheral blood stem cells	6 months	Coronary artery restenosis	PCI/Aspirin+ clopidpgrel, β-blockers, ACE inhibitors	(27)
**Kang, H.-J., et al/ ** **2004**	74	Antero-lateral wall MI	Peripheral blood stem cells	6 months	None	PCI/Aspirin+ clopidpgrel, β-blockers, ACE inhibitors, HMG CoA inhibitor	(27)
**Kang, H.-J., et al/ ** **2004**	63	Anterior septum MI	Peripheral blood stem cells	6 months	Coronary artery restenosis	PCI/Aspirin+ clopidpgrel, β-blockers, ACE inhibitors	(27)
**Kang, H.-J., et al/ ** **2004**	55	Inferior wall MI	Peripheral blood stem cells	6 months	Coronary artery restenosis	PCI/Aspirin+ clopidpgrel, β-blockers, ACE inhibitors, HMG CoA inhibitor	(27)

**Inclusion and exclusion criteria**

Studies reporting the stem cell implantation in human and non-human subjects (mammals) with myocardial damage were included in our study, whereas stem cell transplantation in tissues which were other than cardiac origin was excluded from our review. 

**Frequencies and percentages**

Table 1 **: **Twenty different cases of stem cell transplantation in animal subjects were reviewed. The animals (n=20) consisted of 10 rats (50%), 4 pigs (20%), 3 sheeps (15%), 2 dogs (10%) and 1 rabbit (5%). All mammals were included because of close resemblance of cardiac anatomy to human heart. Induction of MI was performed in all subjects by coronary artery ligation except for two cases in which cryoinjury was performed^   11 ^, while the other one had myocarditis induced by immunization with porcine cardiac myosin^   12 ^. The duration of follow-up varied from 4 weeks to one year. All subjects showed improved cardiac function except one in which no change ^   13 ^ was reported before and after implantation of stem cells. 

Table 2 **: **Data of 24 cases of stem cell transplantation in human subjects are collected in Table.2. Patients were within the age range of 46 to 76 years old. Most of the patients presented with anterior wall (n=8) or inferior wall MI (n=5). All patients received PCI as an initial intervention. The duration of follow-up was varying from 3 months to 10 months. The most frequent complication of stem cell transplantation was coronary artery restenosis (n=5). Moreover, few cases showed minor complications like pneumonia, bronchitis, bleeding from internal mammary artery and bleeding from marrow harvest site.

## Discussion

 Clinical and theoretical studies on evaluation of MSCs have been the topic of ongoing discussion for some decades. Although the pilot studies of autologous mesenchymal precursor cells have shown trophic capacity to regenerate myocardium, many practical and clinical aspects are still controversial. The focus of our review is to assess the physiology and molecular mechanism of stem cell intervention, post-infusion adverse outcomes and failure of therapy. Clinical human trials and experimental studies on mammalian animals reported in past two decades are also part of our discussion. 

**Molecular science of stem cell therapy**

Both animal and clinical trials have provided the evidence of improved cardiac function after MSC transplantation. A comparative study was conducted by  Xinyang Hu  et al. on rats with permanent MI to evaluate the possible mechanism. Normoxic and hypoxic stem cells were injected within endocardium around the peri-infarct region after 30 minutes of MI. In vitro and in vivo assessment of MSC death, angiogenesis, infarct size and cardiac function was observed after 6 weeks of transplantation. Hypoxic stem cell induces pro-survival and pro-angiogenic factors including hypoxia-inducible factor 1, angiopoietin-1, VEGF, Flk-1, erythropoietin, Bcl-2 and Bcl-xL. Cell death and caspase-3 activation in hypoxic group were significantly lower compared to those of in normoxic stem cells both in vitro and in vivo. Transplantation of hypoxic versus normoxic MSCs after MI resulted in increment of angiogenesis as well as enhanced morphologic and functional benefits of stem cell therapy. Mild to moderate degree of hypoxia induces pro-survival and pro-angiogenic factors within the MSCs, contributing to the enhanced tolerance of H-MSCs to apoptosis and increased angiogenesis after transplantation^   14 ^.

It is hypothesized that IGF-1 has a key role in attracting stem cells to damaged tissue, their differentiation via release of paracrine factors and activation of molecular pathway for cell survival .It was observed that ex-vivo over expression of IGF-1 resulted in elevated SDF-1 alpha levels, a potent chemo-attractant of stem cells, both in vitro and after transplantation in the infracted heart^   15 ^. SDF-1 increases expression approximately 3-fold in MSCs which leads to significant reduction in cardiac myocyte death, increased vascular density, and improvement in cardiac function following the intravenous infusion of MSCs in 24 hours after MI^   16 ^. Mesenchymal stem cells express SDF-1; thus their engraftment could sustain myocardial SDF-1 levels in the infarct border zone to a time at which cardiac myocytes express CXCR4 ^16^^,^^17^  and ischemic preconditioning leads to the early expression of CXCR4 by cardiac myocytes^   18 ^. Growth factors, like FGF-2, that signal through phosphatidylinositol kinase-3 can lead to synergistic up-regulation of CXCR4 in the setting of hypoxia ^19^^,^^20^ . SDF-1:CXCR4 axis is not the only pathway active in stem cell based myocardial repair. Interleukin 10^21^, thymosin beta 4^   22 ^ and other non-CXCR effects by variety of growth factors have all been demonstrated the induction of myocardial repair^   23 ^.

Embryonic stem cells (ESCs) hold great promise for cardiac regeneration but are susceptible to various concerns. A study by Mohsin K et al. demonstrated that mouse ESC-derived exosomes (*mES* *Ex*) possess the ability to augment cardiac function in infarcted hearts. *mES Ex* enhanced neovascularization, cardiomyocyte survival and reduced fibrosis after myocardial infarction consistent with resurgence of cardiac proliferative response. Importantly, *mES Ex* augmented cardiac progenitor cell (CPC) survival, proliferation and cardiac commitment concurrent with increased c-kit+ CPCs in vivo 8 weeks after in vivo transfer along with formation of bonafide new cardiomyocytes in the ischemic heart. The underlying basis for the beneficial effect of *mES Ex* was tied to delivery of ESC specific miR-294 to CPCs, promoting increased survival, cell cycle progression and proliferation^   24 ^.

**Outcome of stem cell therapy**

Similar human clinical trial was conducted in 2004, in which left ventricular ejection fraction was assessed after successful PCI. Intracoronary infusion of bone marrow stem cells showed improved LVEF by 0.7% in the control group and 6.7 % in the bone marrow cell group. Transfer of bone marrow cell enhanced left ventricular systolic function in myocardial segments adjacent to infracted area^25^. In vivo studies demonstrated the induction of angiogenesis after BMC transplantation in cryoinjury-derived scar. At 3 weeks after injury, fresh BMCs (n=9), cultured BMCs (n=9), 5-aza-induced BMCs (n=12) and medium (control, n=12) were transplanted into the scar. After 8 weeks of myocardial injury, cardiac-like muscle cells which stained positively for myosin heavy chain and troponin I were observed in the scar tissues of 3 BMC transplanted groups. Only 5-aza-treated BMC transplanted hearts had higher systolic function (P<0.05) than that of the control hearts^   11 ^. 

Similarly, as discussed in Table 1, in our review of literature, hemodynamic status after stem cell therapy was assessed in terms of left ventricular ejection fraction or left ventricular function in mammals. Out of 24 cases, 23 showed an improvement in LVEF or LV function, and only one case study failed to show any improvement in left ventricular function^   13 ^. 

**Safety profile**

In past few decades, stem cell transplantation has emerged as a relatively safe and successful intervention in treating patients with acutely damaged myocardium. But some studies reported minor adverse outcomes related to this therapy. A study conducted by Stamm C et al. reported 6 cases of autologous MSC transplant; of whom 3 developed bronchitis, supra-ventricular tachycardia with pneumonia and bleeding from internal mammary artery^   26 ^. Similarly, hematoma of bone marrow at the harvest site of BSCs was also observed^   10 ^. In a randomized clinical trials on 27 patients, few developed restenosis of coronary artery after intracoronary infusion of peripheral blood stem cells^   27 ^. In vivo studies on mammalian animals also demonstrated some poor outcomes. An experimental trial related to intracoronary infusion of MSCs in dogs provided evidence of acute myocardial ischemia with the induction of micro-infarction^   28 ^. Clinical application of MSC-based therapy is restricted because of the poor survival of implanted cells, and this poor survival remains poorly understood. A study by Mao J showed using a tumor necrosis factor (TNF)-α-induced bone marrow (BM)-MSC injury model in vitro and a rat MI model in vivo, miR-23a was involved in TNF-α-induced BM-MSC apoptosis through regulating caspase-7 and the injection of BM-MSCs over-expressing miR-23a could improve left ventricular (LV) function and reduce infarct size in the rat MI model^   29 ^.

The overall safety profile of stem cell-based therapy is excellent as evident by our case review; only 5 out of 24 cases showed coronary artery restenosis as a result of stem cell transplant (Table 2).

**Challenges faced in stem cell therapy**

Stem cell therapy faces several challenges. It is difficult to grow, preserve, and transport stem cells before they are administered to the patient. Synthetic analogs for stem cells represent a new approach to overcome these hurdles. In a study by Lan Luo et al., they successfully fabricated a synthetic MSC (syn-MSC) therapeutic particle and demonstrated its regenerative potential in mice with acute myocardial infarction. They packaged secreted factors from human bone marrow–derived mesenchymal stem cells (MSC) into poly (lactic-co-glycolic acid) micro-particles, and then coated them with MSC membranes. Syn-MSC exhibited a factor release profile and surface antigens similar to those of genuine MSC. Syn-MSC promoted cardiomyocyte functions and displayed cryopreservation and lyophilization stability in vitro and in vivo. In a mouse model of acute myocardial infarction, direct injection of syn-MSC promoted angiogenesis and mitigated left ventricle remodeling^   30 ^.

Similar studies conducted by Reinecke H et al. believed that the fundamental reason of transplantation failure is the delay in seeking medical attention^   31 ^. Hematopoietic stem cells (HSCs) possess the greatest capacity of transdifferentiation into multiple cell lineages. Despite the fact, another study by Reinecke H et al. reported the failure of hematopoitic stem cell therapy in 175 subjects^   32 ^. Such failure raises concerns about stem cell therapy which requires further investigation to establish an evidential consensus about its therapeutic efficacy.

**Ethical consideration**

Ethics in stem cell therapy is another vital component of modern medicine. It is a topic of immense value. We have discussed some common ethical aspects faced during stem cell-based therapy. A research by Anne M in 2001 highlighted the point that using fetal stem cell might be an unethical because fetus cannot provide informed consent which is required for the extraction of stem cells.^   33 ^. 

According to new FDA guidelines for stem cell-based therapy released in 2006, each patient should be informed about the risk of acquiring any infections or genetic from donor tissue, rejection of implanted tissue, contamination or damage to the donor tissue, purity and potency of stem cells, safety and effectiveness in vivo of stem cell therapy^   34 ^.

## CONCLUSION

 From review of evidence, we observed that induction of angiogenesis, prolonged cell survival and paracrine factors are essential for successful stem cell transplantation. Most frequent patient- related adverse outcome was restenosis of coronary vessels, but the overall safety profile was significant. Human trials conducted in past few years showed as remarkable improvement in overall cardiac functioning as experimental trials in different mammals. Long-term success rate of MSCs therapy is debatable as some failures of intervention were also reported in medical literature. Ethically, embryonic stem cell transplantation is the most controversial topic which requires a detailed assessment.

## Abbreviation

AM=Adrenomedullin

Bcl-2=B- Cell Lymphoma 2

Bcl-xL= B-Cell Lymphoma-extra large

BM-MNC=Bone marrow mononuclear cells

CPC=cardiac progenitor cell

CXCR4 =C-X- C chemokine receptor type 4

EC=Endocardial

EDCs=Explant derived cardiac stem cells

EGFP=enhanced green fluorescent protein

EPC=Endothelial progenitor cells

Flk-1=a receptor for VEGF

hMSCs=Human mesenchymal stem cells

IC=intracoronary

IGF1=Insulin like growth factor

IV=intravenous

LAD=left anterior descending artery

LVEDP=Left ventricular end diastolic pressure

LVEF=Left ventricular ejection fraction

LVSP=Left ventricular systolic pressure

mES Ex=mouse ESC derived exosomes

MNC=bone marrow derived mononuclear cells

MPC=Mesenchymal precursor cell

SDF-1=stromal cell derived factor 1

SynMSC =synthetic MSC

TNF-α= tumor necrosis factor

VEGF=vascular endothelial growth factor
